# Identification of novel tumor-associated antigens and evaluation of a panel of autoantibodies in detecting oral cancer

**DOI:** 10.1186/s12885-023-11247-w

**Published:** 2023-08-28

**Authors:** Weihong Xie, Guiying Sun, Junfen Xia, Huili Chen, Chen Wang, Juan Lin, Peng Wang

**Affiliations:** 1 Department of Oral and Maxillofacial Surgery, The First Affiliated Hospital of Zhengzhou University, Zhengzhou University, Zhengzhou, Henan Province 450052 China; 2 Department of Epidemiology and Statistics, College of Public Health, Zhengzhou University, Zhengzhou, Henan Province 450001 China; 3 Henan Key Laboratory of Tumor Epidemiology, Zhengzhou University, Zhengzhou, Henan Province 450052 China; 4 Office of Health Care, The Third Affiliated Hospital of Zhengzhou University, Zhengzhou University, Zhengzhou, Henan Province 450052 China; 5 Academy of Medical Science, Zhengzhou University, Zhengzhou, Henan Province 450052 China

**Keywords:** Autoantibody, Bioinformatics, Early detection of Cancer, Logistic model, Oral cancer

## Abstract

**Background:**

We aimed to identify tumor-associated antigen (TAA) biomarkers through bioinformatic analysis and experimental verification, and to evaluate a panel of autoantibodies against tumor-associated antigens (TAAbs) for the detection of oral cancer (OC).

**Methods:**

GEO and TCGA databases were used to screen significantly up-regulated genes related to OC, and protein-protein interaction (PPI) analysis and Cystoscope software were used to identify key genes. Enzyme-linked immunosorbent assay (ELISA) was used to detect the expression levels of autoantibodies in 173 OC patients and 173 normal controls, and binary logistic regression analysis was used to build a diagnostic model.

**Results:**

Using bioinformatics, we identified 10 key genes (AURKA, AURKB, CXCL8, CXCL10, COL1A1, FN1, FOXM1, MMP9, SPP1 and UBE2C) that were highly expressed in OC. Three autoantibodies (anti-AURKA, anti-CXCL10, anti-FOXM1) were proven to have diagnostic value for OC in the verification set and the validation set. The combined assessment of these three autoantibodies improved the diagnostic value for OC, with an area under the curve (AUC), sensitivity and specificity of 0.741(95%CI:0.690–0.793),58.4% and 80.4%, respectively. In addition, the combination of these three autoantibodies also had high diagnostic value for oral squamous cell carcinoma (OSCC), with an AUC, sensitivity and specificity of 0.731(95%CI:0.674,0.786), 53.8% and 82.1%, respectively.

**Conclusions:**

Our study revealed that AURKA, CXCL10 and FOXM1 may be potential biomarkers and the panel of three autoantibodies (anti-AURKA, anti-CXCL10 and anti-FOXM1) had good diagnostic value for OC.

## Introduction

Global Cancer Statistics 2020 report noted 377,713 new global cases and 177,757 new oral cancer (OC)-related deaths [1]. Oral squamous cell carcinoma (OSCC) accounts for ≥ 90% of OC cases [2]. Although surgical resection combined with chemotherapy and radiotherapy improve survival, the overall 5-year survival rate of OC patients within the past 20 years is still less than 50% [3]. A large part of the reason for the low survival rate is the delay in diagnosis, and OC is easily confused with a benign disease such as oral ulcer at the initial stage of the disease [4]. If timely treatment can be provided at the initial stage of OC, the 5-year survival rate will be higher than 90% [5]. Therefore, timely diagnosis plays a crucial role in improving the survival of patients with OC.

Biopsy-based pathology is the gold standard for the diagnosis of OC, but it is invasive and requires professional technical training, so it incurs high economic costs. Brush biopsy or toluidine blue staining can provide early diagnosis, but the difficult-to-access locations of some tumors limit the reliability of these methods in clinical applications. CT addresses these shortcomings to a large extent, but CT should not be used as a routine examination method, because it has strong radiation to the human body and low sensitivity. At present, no reliable auxiliary examinations have been found to replace tissue biopsy and histological evaluation [6]. Therefore, we should devote ourselves to exploring a minimally invasive, sensitive and economical method to diagnose OC. In recent years, molecular technologies have attracted increasing attention. Research on salivary biomarkers is gradually increasing, but the methodological problems of oral carcinogenesis and heterogeneity within the tumor limit their interpretation. Liu et al. [7] reported that inflammatory plasma proteins can be used as potential biomarkers in patients with OSCC.

Research in the field of molecular markers shows that autoantibodies against tumor- associated antigen (TAAbs) in serum may be detected a few months before the onset of symptomatic cancer. In addition, TAAbs have the advantages of being able to exist in the patient’s blood for a long time, being easy to detect, and the detection process being minimally invasive to the patient, and may be used as new diagnostic biomarkers for early cancer patients [8–10]. TAAb may be helpful for the early diagnosis of various solid tumors, such as esophageal cancer [11], lung cancer [12], breast cancer [13], hepatocellular carcinoma [14], colorectal cancer [15], thyroid cancer [16] and gastric cancer [17] and so on. Compared to the study of autoantibodies in the diagnosis of other cancers, the study of autoantibodies in the detection of OSCC is limited.

In recent years, differentially expressed genes (DEGs) and enrichment analysis using microarray and bioinformatics techniques have become fast and effective methods to discover biomarkers. In this study, differential analysis was conducted based on the datasets related to OC in TCGA and GEO databases, and protein-protein interaction (PPI) analysis was conducted to identify key genes. The proteins encoded by these genes were used as potential TAAs, and enzyme-linked immunosorbent assay (ELISA) was used to verify the expression levels of the corresponding autoantibodies in OC and normal control (NC) to provide evidence supporting the use of TAAbs as biomarkers for the diagnosis of OC.

## Materials and methods

### Differentially expressed gene analysis

The GEO microarray sets (GSE31056 and GSE37991) containing OSCC and non-tumor samples were obtained from the National Center for Biotechnology Information (NCBI) (GEO, https://www.ncbi.nlm.nih.gov/geo/). The DEGs in the two GEO datasets were screened online using by GEO2R. Simultaneously, based on TCGA-head and neck squamous cell carcinoma (HNSC) and Genotype-Tissue Expression (GTEx) databases, we also used the GEPIA (http://gepia2.cancer-pku.cn/#degenes) web server to screen DEGs using the LIMMA method. |logFC|> 1and adjusted *P* value < 0.05 were considered statistically significant for DEGs. A volcano map of the DEGs was drawn using the SangerBox tools (http://www.sangerbox.com/tool). Subsequently, we intersected the up-regulated DEGs from the three datasets using a Venn diagram.

### Functional annotation and identification of hub genes

Gene Ontology (GO) and Kyoto Encyclopedia of Genes and Genomes (KEGG) analyses were performed and a PPI network was constructed using the retrieval of interacting genes (STRING) database (https://string-db.org/) (version 11.5). The results of GO analysis and KEGG analysis were visualized using the Sangerbox web tool(http://www.sangerbox.com/tool). The confidence score for the PPI analysis was set at > 0.4. The protein interaction data were imported into the Cytoscape software (version 3.7.2, http://www.cytoscape.org). We used the degree topological algorithm of the cytoHubba plugin to select the top 10 genes in the network as hub genes and visualized them using Cytoscape.

### Enzyme-linked immunosorbent assay

Using bioinformatics methods, 10 hub genes (AURKA, AURKB, CXCL8, CXCL10, COL1A1, FN1, FOXM1, MMP9, SPP1 and UBE2C) were found to be highly expressed in patients with oral cancer. To verify whether the proteins encoded by these key genes can produce corresponding autoantibodies, we further detected the expression levels of autoantibodies corresponding to these 10 proteins in the serum of 173 OC patients and 173 NCs using ELISA. Because of the problem of protein purification, the purity and concentration required by the experiment could not be achieved. therefore, we did not purchase the appropriate purified recombinant protein AURKB, and we finally purchased nine recombinant proteins from CLOUD-CLONE CORP (Wuhan, China). The nine recombinant proteins (AURKA, CXCL8, CXCL10, COL1A1, FN1, FOXM1, MMP9, SPP1, and UBE2C) were diluted in carbonate buffer (pH = 9.6) to a concentration of 0.250 µg/mL. All the diluted proteins were coated onto 96-well plates overnight at 4 °C; and then the plates were blocked with 2% bovine serum albumin (BSA) in phosphate-buffered saline containing 0.05% Tween-20 (PBST) overnight at 4 °C. After washing with PBST, serum with dilution of 1:100 was added to each well except the wells for blank wells. The plates were then placed in water baths at 37 °C for 1 h, followed by washing with PBST. The plates were incubated with horseradish peroxidase (HRP)-conjugated goat anti–human IgG (Wuhan Aoko Biotechnology Co. Ltd.) at 1:5000 dilution in 1% BSA, in 37 °C water baths for 1 h. A solution of 3,3ʹ,5,5ʹ-tetramethyl benzidine (TMB)-H_2_O_2_-urea was used as the detecting agent and 50µL of 2 M sulfuric acid was added to each well to stop the reaction. The optical density (OD) was measured at 450 and 620 nm using a multilabel plate reader (PerkinElmer). The difference between OD450 and OD620 was subsequently analyzed.

### Study population

In this study, 173 patients with OC and 173 NC were recruited from a third-level grade A hospital in Henan Province. Sixty patients with OC and 60 controls were randomly assigned to the verification set, and 113 patients with OC and 113 controls were randomly assigned to the validation set. All patients in this study were confirmed by pathological examination, besides, none of them received any treatment before blood collection. In the NCs, individuals with autoimmune diseases and oral diseases were excluded. Frequency matching of sex and age was performed between the case and control groups. This study was approved by the Medical Ethics Committee of Zhengzhou University (ZZURIB 2019-001), and conformed to the standards set by the Declaration of Helsinki. All subjects have signed the informed consent form.

### Statistical analysis

IBM SPSS 25.0 and GraphPad Prism 9.1.1 were used in the study. All statistical analyses were based on a two-tailed test, and *P* < 0.05 was considered statistically significant. The nonparametric test was used to compare the expression levels of autoantibodies between patients with OC and NCs. The OD value corresponding to the maximum Youden’s index when the specificity was greater than 80% was determined as the cut-off value. The receiver operating characteristic (ROC) curve was used to evaluate the diagnostic value of the autoantibodies in the different groups. The area under the ROC curve (AUC), sensitivity, specificity, accuracy, and Youden’s index (YI) were calculated to estimate the diagnostic value of these autoantibodies.

## Results

### Identification of DEGs

**Fig. 1:**
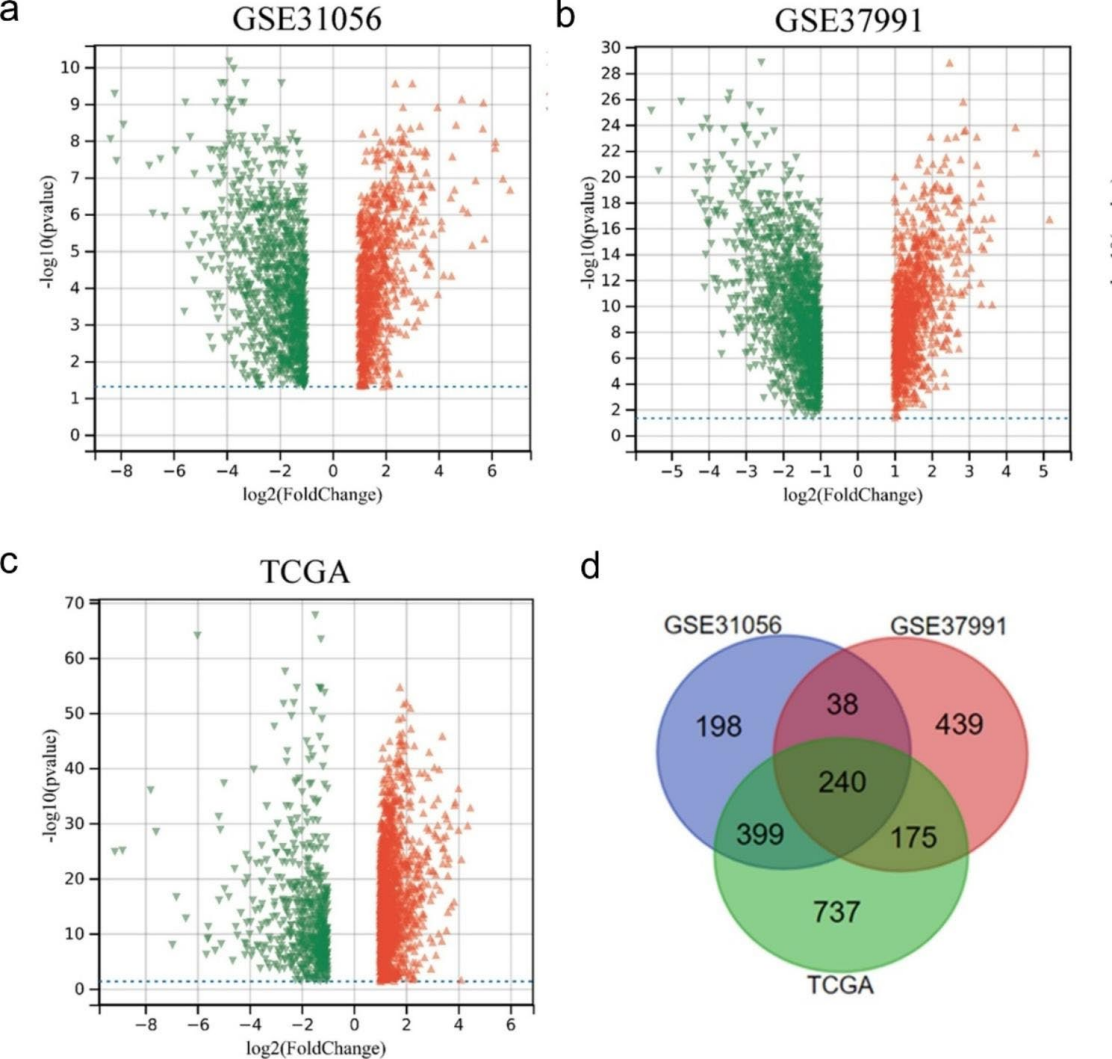
Differential expression gene analysis and Venn diagram. (**a**) Volcano map of differentially expressed genes in GSE31056 dataset. (**b**) Volcano map of differentially expressed genes in GSE37991 dataset. (**c**) Volcano map of differentially expressed genes in TCGA dataset. (**d**) Intersection of differentially up-regulated genes in three datasets. The red triangles represent differentially up-regulated genes, while the green triangles represent differentially downregulated genes

Only up-regulated DEGs were chosen for the following study in this research. Based on the cut-off criteria (|log fold change (FC)| > 1.0 and *P* < 0.05), compared with normal tissues, the GSE31056 dataset included 875 up-regulated DEGs, GSE37991 database included 892 up-regulated DEGs, and TCGA database included 1551 up-regulated DEGs (**Fig. 1a-c**). A total of 240 up-regulated DEGs were significantly expressed among all three datasets (**Fig. 1d**).

### Functional annotation and identified potential hub genes

**Fig. 2:**
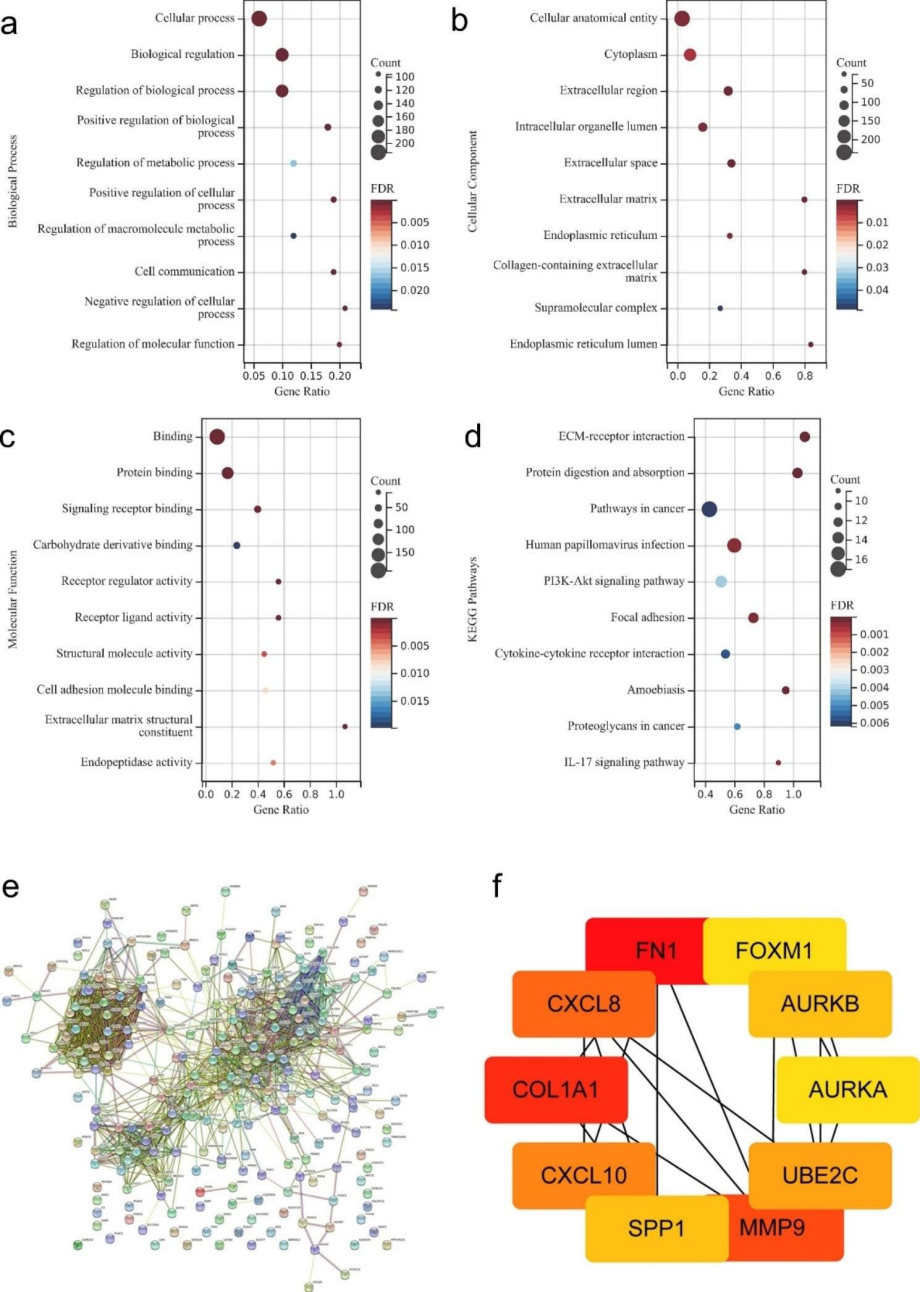
Functional annotation and identification of hub genes via the PPI network

The most enriched GO terms in the biological process (BP), cellular component (CC) and molecular function (MF) categories were shown in **Fig. 2a-c**. In the BP category, a high number of DEGs were associated with cellular processes, biological regulation, and the regulation of biological processes. In the CC category, the DEGs were remarkably related to the cellular anatomical entity, cytoplasm and extracellular space. MF analysis revealed that the DEGs were mainly related to binding, protein binding and signaling receptor binding. KEGG analysis revealed that the extracellular matrix-receptor interaction, protein digestion and absorption, and pathways in cancer were mainly enriched signaling pathways (**Fig. 2d**). The up-regulated genes were input into STRING to construct the PPI network (**Fig. 2e**). The hub genes were sequenced according to their degree values. Next, the PPI network of the hub genes was constructed and showed that the genes strongly interacted with each other (**Fig. 2f**).

### Characteristics of study participants

The expression levels of TAAs obtained by bioinformatics methods in OC patients and the NC group were verified by ELISA. Serum samples from 173 OC patients and 173 NCs were used for ELISA. Detailed clinical information of the 346 participants is presented in **Table 1**. In both the verification set and validation set, there was no significant difference in sex (*P* = 0.251, *P* = 0.361) or age (*P* = 0.873, *P* = 0.960) distribution between the OC patients and the NCs group.

Significant enrichment terms of (**a**) biological process, (**b**) cellular component, (**c**) molecular function, and (**d**) KEGG signaling pathway of up-regulated DEGs. (**e**) PPI network of DEGs analyzed using the STRING database. (**f**) Detection of hub genes in the PPI network of common DEGs. The highlighted ten genes were AURKA, AURKB, CXCL8, CXCL10, COL1A1, FN1, FOXM1, MMP9, SPP1 and UBE2C.GO, gene ontology; DEGs, differentially expressed genes; KEGG, Kyoto Encyclopedia of Genes and Genomes; OC, oral cancer.

**Table 1:** Characteristics of study participants

Variables	Verification set		Validation set		
	OC(n = 60)	NC(n = 60)	OC(n = 113)	NC(n = 113)	
Gender					
Male, n (%)	36(60.1)	42(70.0)	81(71.7)	74(65.5)	
Female, n (%)	24(39.9)	18(30.0)	32(28.3)	39(34.5)	
Age, years, Mean ± SD	58.47±12.26	57.87±13.87	57.12±13.08	53.53±11.88	
Cancer type					
OSCC	46(76.7)		86(76.1)		
Non-OSCC	14(23.3)		27(23.9)		
Tumor location, n (%)					
Oral cavity	51(85.0)		87(77.0)		
Oropharynx	9(15.0)		21(18.6)		
Unknow	0(0.0)		5(4.4)		
Family tumor history, n (%)					
Yes	4(6.7)		8(7.1)		
No	46(76.6)		92(81.4)		
Unknow	10(16.7)		13(11.5)		
Histological grade, n (%)					
High	26(43.3)		50(44.2)		
Medium	15(25.0)		36(31.9)		
Low	6(10.0)		15(13.3)		
Unknown	13(21.7)		12(10.6)		
TNM stage, n (%)					
I	20(33.3)		29(25.8)		
II	15(25.0)		36(31.8)		
III	11(18.4)		20(17.7)		
IV	9(15.0)		25(22.1)		
Unknown	5(8.3)		3(2.6)		
Lymph node metastasis, n (%)					
Positive	12(20.0)		33(29.2)		
Negative	44(73.3)		76(67.3)		
Unknown	4(6.7)		4(3.5)		

Abbreviations: OC, oral cancer; NC, normal control; OSCC, oral squamous cell carcinoma

### Diagnostic value of single autoantibody in OC by ELISA

**Fig. 3:**
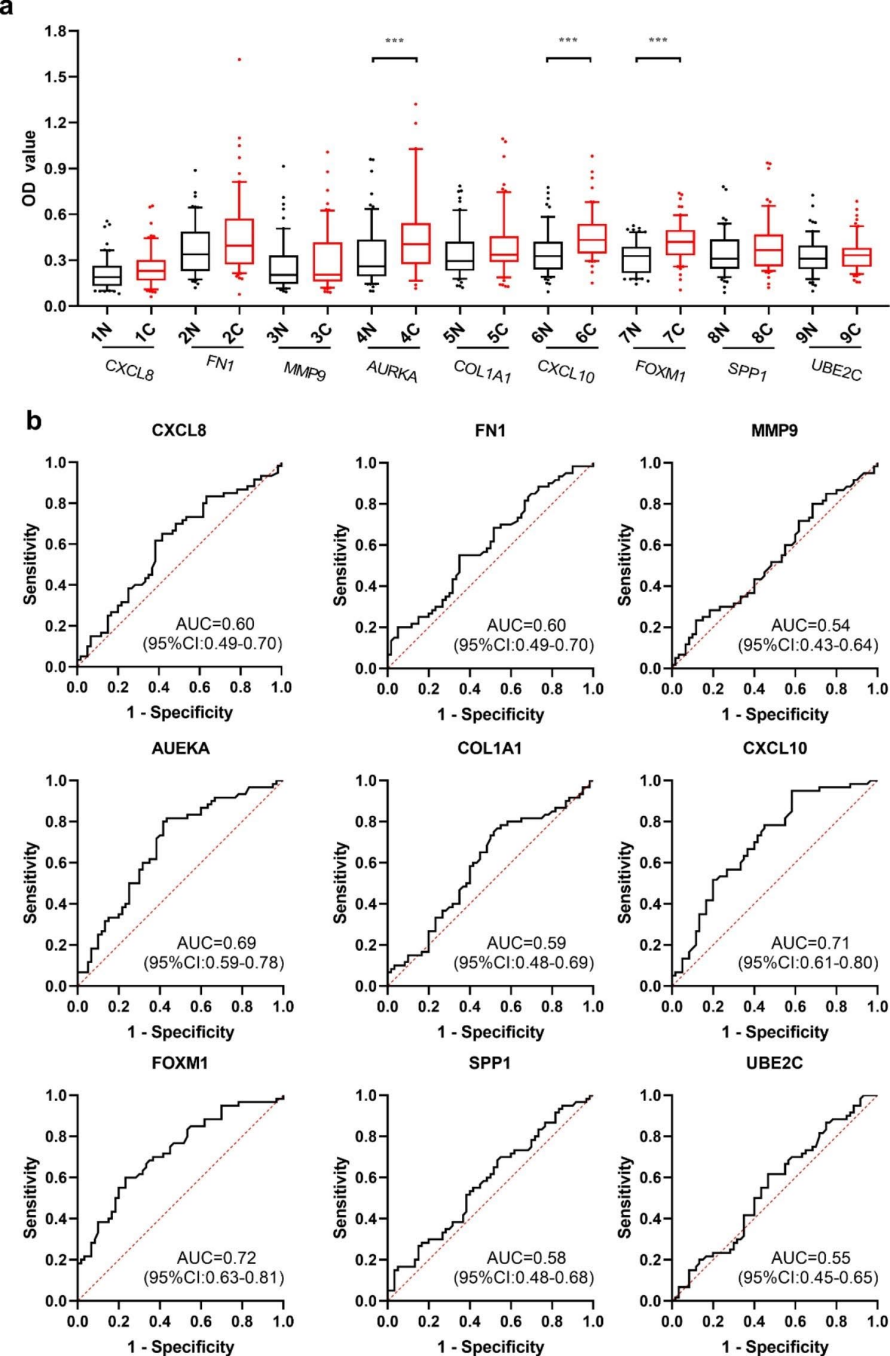
Expression level and ROC curve analysis of nine autoantibodies in patients with OC and NC in the verification set. (**a**) Serum levels (optical density, OD) of nine autoantibodies in patients with OC and NC; C (N = 60); N (N = 60). (**b**) ROCs of distinguishing between OC and NC for nine TAAbs. AUC, area under the curve; C, cancer; CI, confidence interval; N, normal; NC, normal control; OC, oral cancer; ROC, receiver operating characteristic; TAAb, anti-tumor associated antigen autoantibody. ***P < 0.001

**Fig. 4:**
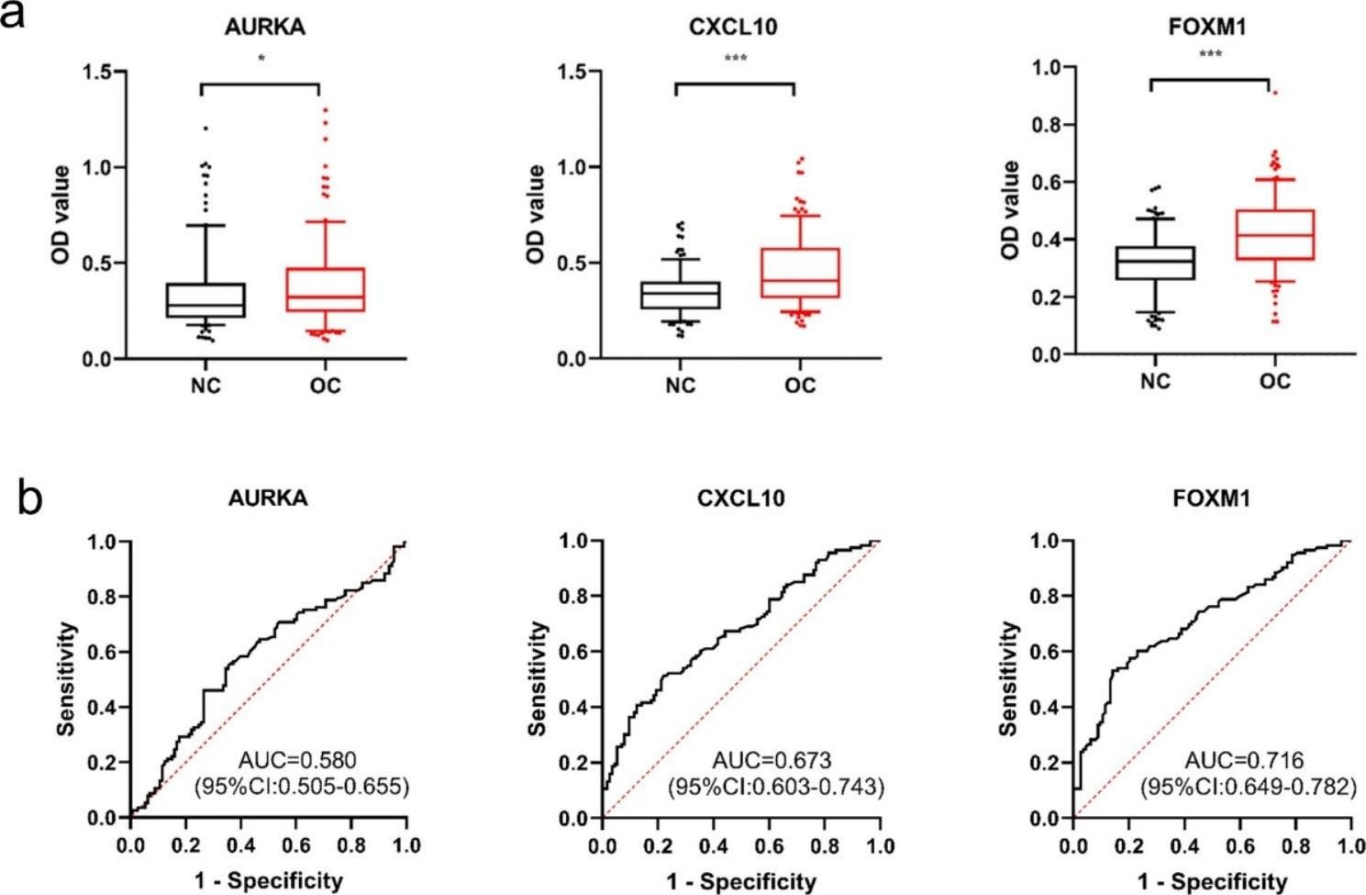
Expression level and ROC curve analysis of three autoantibodies in patients with OC and NC in the validation set. (**a**) Serum levels (optical density, OD) of nine autoantibodies in patients with OC and NC; OC (N = 113); NC (N = 113). (**b**) ROCs of distinguishing between OC and NC for three TAAbs. AUC, area under the curve; C, cancer; CI, confidence interval; N, normal; NC, normal control; OC, oral cancer; ROC, receiver operating characteristic; TAAb, anti-tumor associated antigen autoantibody. ***P < 0.001; *P < 0.05

**Fig. 5:**
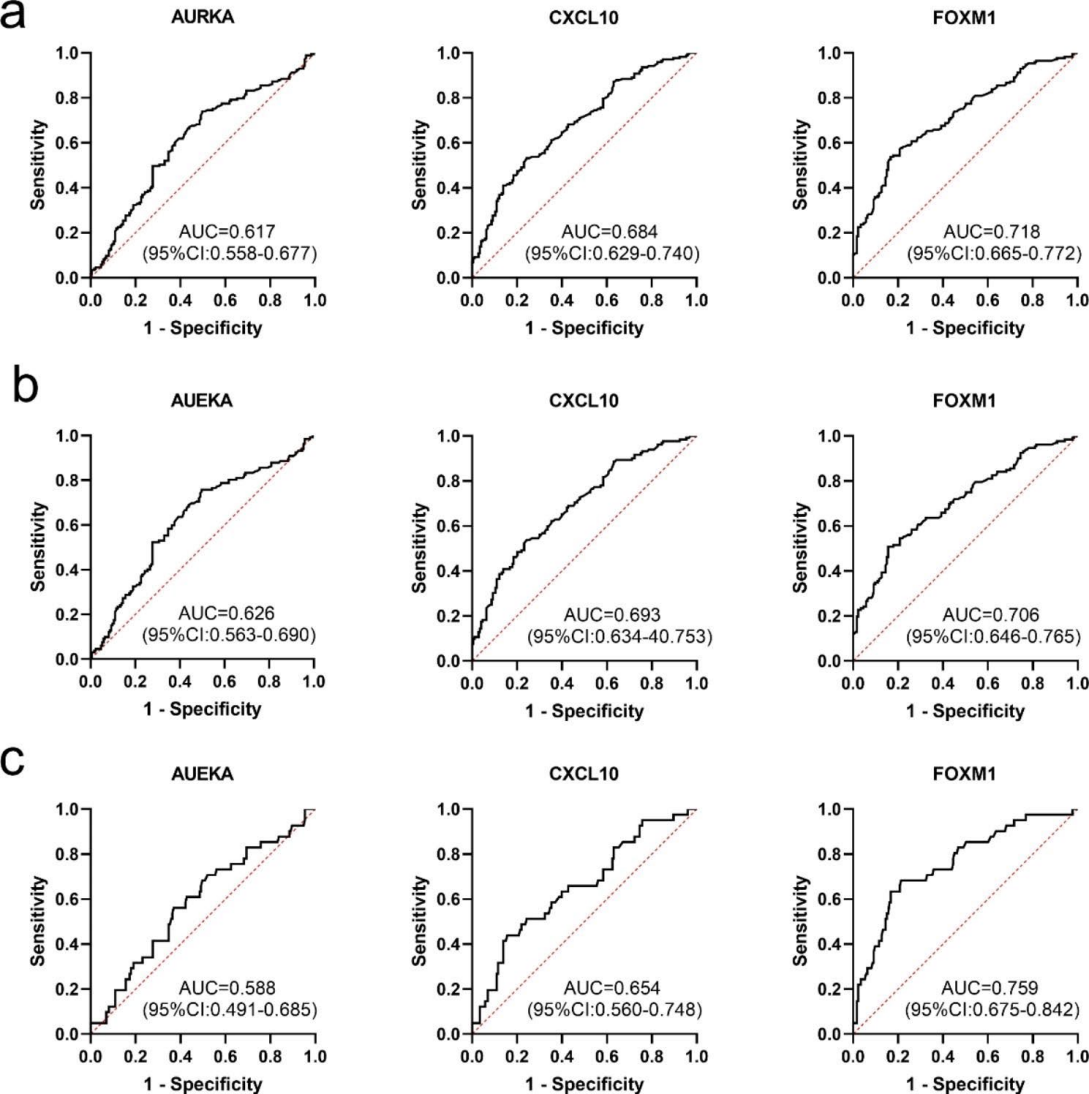
ROC analysis of three autoantibodies for detecting OC. (**a**) Diagnostic value of three autoantibodies for OC. (**b**) Diagnostic value of three autoantibodies for OSCC. (**c**) Diagnostic value of three autoantibodies for non-OSCC. AUC, area under the curve; CI, confidence interval; OSCC, oral squamous cell carcinoma

Ten TAAs were selected as biomarkers for bioinformatic analysis. Except for the AURKB recombinant protein, which did not meet the experimental conditions, the selected recombinant TAA proteins (AURKA, COL1A1, CXCL8, CXCL10, FN1, FOXM1, MMP9, SPP1 and UBE2C) were used as coating antigens to detect the corresponding autoantibodies in sera in the verification set. ELISA results in the verification set showed that the levels of anti-AURKA, anti-CXCL10 and anti- FOXM1 autoantibodies in the OC group were significantly higher than in those in the NC group (**Fig. 3a**). The AUC of a single anti-TAA autoantibody ranged from 0.686 to 0.724 in the detection of OC (**Fig. 3b**). We further verified the diagnostic value of these three anti-TAA autoantibodies in the validation set by ELISA and found that the levels of anti- AURKA, anti-CXCL10 and anti- FOXM1 autoantibodies in the OC group were also significantly higher than those in the NC group (**Fig. 4a**). These three anti-TAA autoantibodies also had diagnostic value for OC in the validation set (**Fig. 4b**). When the two groups of cases were combined as one group and the two groups of controls were combined into one group, these three autoantibodies (anti-AURKA, anti-CXCL10 and anti- FOXM1 autoantibodies) have diagnostic value for OC, with AUCs, sensitivity, specificity of 0.617,32.4%,80.9% and 0.684,45.7%,81.5% and 0.718,54.3%,80.4%, respectively (**Fig. 5a**; **Table 2**). When the patients with OC were classified according to histological types, these three indicators had diagnostic value for OSCC, and the AUC of these three autoantibodies ranged from 0.626 to 0.706 (**Fig. 5b**). The anti-CXCL10 and anti-FOXM1 autoantibodies were also suggested to be diagnostic antibodies for non-OSCC, with AUCs of 0.654 and 0.759, respectively. The anti-AURKA autoantibody had no diagnostic value for non-OSCC (**Fig. 5c**).

### Construction and evaluation of the diagnostic mode

**Fig. 6:**
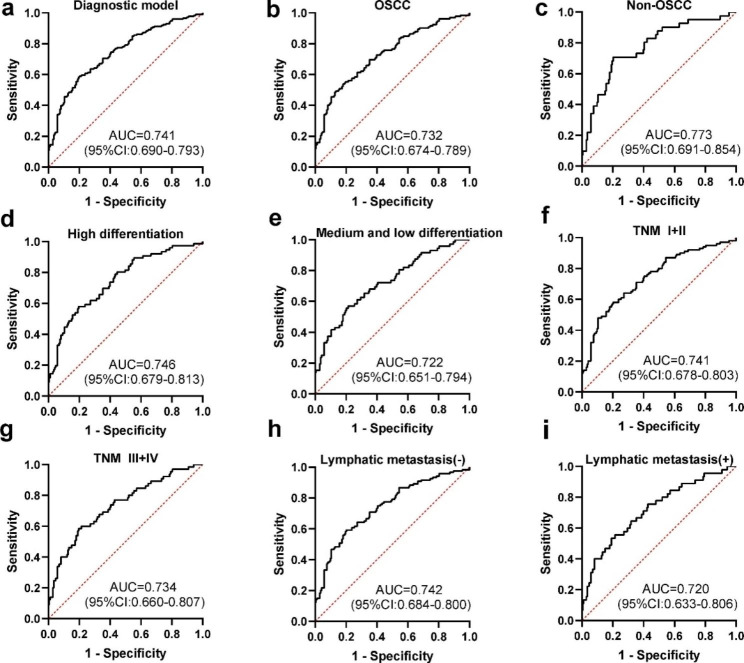
ROC analysis of the model for OC with different clinical characteristics (**a**) ROC analysis of the model for the diagnosis of OC and NC. (**b**-**i**) The diagnostic value of the diagnostic model in different clinical characteristics of OC. AUC, area under the curve; CI, confidence interval; OC, oral cancer; OSCC, oral squamous cell carcinoma, ROC, receiver operating characteristic

No matter the verification set or the validation set, anti-AURKA, anti- CXCL10 and anti- FOXM1 autoantibodies have diagnostic value for OC. To improve the diagnostic value of OC, we further explored the diagnostic performance of the combined autoantibodies. We used the expression levels of 173 OC patients and 173 NCs for anti-AURKA, anti-CXCL10 and anti-FOXM1 autoantibodies to construct a diagnostic model using binary logistic regression analysis. The formula for the diagnostic model was as follows: P = 1/ (1 + EXP (−(− 2.878–1.775×anti-AURKA + 2.306 × anti-CXCL10 + 7.146 × anti-FOXM1))). The AUC of the model that included the three autoantibodies reached 0.741 in the detection of OC, with the sensitivity of 58.4% and the specificity of 80.4% (**Table 2**; **Fig. 6a**).

**Table 2:** Diagnostic value of single autoantibody and the panel of 3 autoantibodies for OC with different clinical characteristics

Variables	n	Se (%)	Sp(%)	YI	Accuracy (%)	P
Single autoantibody						
AURKA	173	32.4	80.9	0.133	56.7	-
CXCL10	173	45.7	81.5	0.272	63.6	-
FOXM1	173	54.3	80.4	0.347	67.4	
Panel of autoantibodies						-
All stage	173	58.4	80.4	0.387	69.4	
TNM I-II	100	57.0	80.4	0.374	56.7	0.886
TNM III-IV	65	58.5	80.4	0.388	51.2	
Lymph node (-)	120	58.3	80.4	0.387	60.4	0.675
Lymph node (+)	45	53.3	80.9	0.343	47.4	
Well differentiated	76	57.9	80.4	0.382	52.9	0.634
Moderately and poorly differentiation	72	54.2	80.4	0.345	51.4	
OSCC	132	53.8	82.1	0.359	61.6	0.418
Non-OSCC	41	68.3	80.4	0.486	48.3	

Abbreviations: OC, oral cancer; Se, sensitivity; Sp, specificity; YI, Youden Index, OSCC, oral squamous cell carcinoma

Patients with OC were stratified according to the clinical characteristics of tumor type, differentiation, tumor stage and lymph node metastasis. ROC analysis was performed for each subgroup and the NC group. The results showed that there were significant differences between OC and NC in each subgroup based on the diagnostic model (**Fig. 6b-i**). The AUCs in OSCC, non-OSCC, high differentiation, medium and low differentiated, early stage (TNM I and II), advanced stage (TNM III and IV), lymph node metastasis (-) and lymph node metastasis (+) groups were all higher than 0.700 (0.732, 0.773, 0.746, 0.722, 0.741, 0.734, 0.742, 0.720, respectively). The diagnostic value of this model for early OC was slightly higher than that for late OC (accuracy rate:58.7% vs. 51.2%). Detailed results were presented in **Table 2**. Compared with those of a single TAA, the sensitivity and accuracy of the model for diagnosing AUC of OC were improved to a certain extent. The DeLong test showed no significant difference in the AUC values among clinical subgroups (*P* > 0.05).

## Discussion

An increasing number of studies have shown that autoantibodies play an important role in the diagnosis and prognosis prediction of many types of cancer [18, 19]. The early CDT lung test, which measured seven autoantibodies associated with lung cancer, has been proven to be helpful in predicting the risk of lung cancer [12, 20]. In this study, we conducted a series of bioinformatics analyses based on TCGA and GEO data, and identified 10 key genes (CXCL8, FN1, MMP9, AURKA, AURKB, COL1A1, CXCL10, FOXM1, SPP1, UBE2C) that were significantly overexpressed in OC, and the proteins encoded by these genes were used as potential biomarkers. Only proteins that can produce immune reactions are called tumor-associated antigens, and the corresponding antibodies are called tumor-associated antigen autoantibodies [21]. We further detected the level of autoantibodies in the sera of patients with OC and NCs using ELISA, and finally we obtained three autoantibodies (anti-AURKA, anti-CXCL10, and anti-FOXM1) with high diagnostic value for OC. The combination of these three autoantibodies improved the diagnostic value of OC, with an AUC, sensitivity and specificity of 0.741(95%CI:0.690–0.793),58.4% and 80.4%, respectively. The panel also had high diagnostic value for early OC, and the AUC, sensitivity and specificity were 0.741(95%CI:0.678–0.803), 57.0%, and 80.4%, respectively. To the best of our knowledge, this is the first report on three autoantibodies in OC. In this study, three novel TAAbs (anti-AURKA, anti-CXCL10, and anti-FOXM1) with diagnostic value for OSCC were identified with AUCs of 0.626, 0.693 and 0.706, respectively. In addition, the combination of these three autoantibodies also has high diagnostic value for OSCC, with an AUC, sensitivity and specificity of 0.732(95%CI:0.674,0.789), 53.8% and 82.1%, respectively.

To date, there have been few studies on autoantibodies as diagnostic markers of OC. In 1998, Ralhan et al. [22] confirmed that P53 protein can cause the immune response of patients with OC through ELISA, and found that anti-P53 autoantibody may be a diagnostic biomarker of OC. Subsequently, research by Porrini et al. [23] also showed that the expression level of p53 autoantibody in the serum of patients with OSCC was higher than that of NCs. Chang et al. [24] and Eto et al. [25] indicated that anti-survivin autoantibody may be a useful noninvasive marker for the diagnosis of head and neck cancer. Giresused et al. [26] used autoantibody-mediated antigen experiments to find that a strong prevalence of anti-CK8 autoantibody occurred in the serum of cancer patients at the early stage of head and neck squamous cell carcinoma. The latest report was that in 2016, Jiang et al. [27] found that anti-MMP7 autoantibody has the ability to differentiate OSCC from NC, and may be used as a biomarker of poor prognosis. These studies have reported the diagnostic value of a single autoantibody for OC. The diagnostic value was limited, and the source of research indicators was not clear. This study verified the diagnostic performance of anti-AURKA, anti-CXCL10, and anti-FOXM1 autoantibodies through a series of bioinformatics analysis and two-stage ELISA, and built a diagnostic model with high value based on logistic regression. This also provides new ideas for subsequent research.

The protein encoded by AURKA is a cell cycle-regulated kinase that may play a role in the development and progression of tumors. A study demonstrated that AURKA contributed to the progression of OSCC by modulating epithelial-to-mesenchymal transition (EMT) and apoptosis via the regulation of ROS [28]. Hiroshi’s study [29] suggested that AURKA played a key role in the growth of OSCC cells, and that targeting AURKA may be a useful therapeutic strategy for OSCC. In addition, in this study, when OC was classified according to histological type, anti-AURKA autoantibodies had a higher diagnostic value for OSCC, suggesting that AURKA may be a diagnostic marker for OSCC. CXCL10 encodes chemokines of the CXC subfamily and ligands of the CXCR3 receptor. Li [30] reported that CXCL10 can promote the proliferation, migration and invasion of OC cells, which may indicate that CXCL10 was related to the occurrence and development of OSCC. FOXM1 gene encodes protein that is transcriptional activators involved in cell proliferation, and are phosphorylated in the M phase and regulates the expression of cell cycle genes. In vitro experiments showed that FOXM1 knockdown reduced the proliferation, migration and invasion of OSCC, and it potentially participated in the epithelial mesenchymal transformation of OSCC cells [31]. One of the limitations of our study was that the diagnostic model was not used in combination with traditional biomarkers (such as CEA and CA199) for the diagnosis of OC.

## Conclusions

In this study, three autoantibodies (anti-AURKA, anti-CXCL10, anti-FOXM1) with good diagnostic performance for OC were identified for the first-time using bioinformatics combined with experimental verification methods, and a diagnostic model of autoantibodies of OC in serum was constructed. The results of this study are encouraging and provide a theoretical basis for the minimally invasive diagnosis of OC. Further validation by prospective cohort is needed to assess the usefulness of the autoantibody panel screening for OSCC in the population. Another focus is to screen related-OC autoantibodies using the human proteome microarray, and further combine the traditional tumor markers commonly used in clinic to obtain higher diagnostic value. The molecular function of their corresponding proteins in the development of OC will be assessed in our next study.

## Data Availability

The datasets generated during and/or analyzed during the current study are available from the corresponding author upon reasonable request.
